# How Relevant Is Sexual Transmission of Zika Virus?

**DOI:** 10.1371/journal.pmed.1002157

**Published:** 2016-10-25

**Authors:** Christian L. Althaus, Nicola Low

**Affiliations:** Institute of Social and Preventive Medicine, University of Bern, Bern, Switzerland

## Abstract

Christian Althaus and Nicola Low reflect on the contribution of sexual transmission to the spread of Zika virus.

Sexual transmission of Zika virus could be as important as transmission through bites from infected mosquitoes, if media attention is any indicator [1,2]. One article argues that “sexual transmission is likely to be a significant contributor to the Zika virus’s spread” [3]. The level of attention in news media is high in the United States and Europe, where most Zika virus infections have been detected in travellers returning from endemic areas. Epidemiologists have inferred transmission through sexual intercourse when sexual partners who have not travelled develop symptoms and are found to have Zika virus in blood samples. Each case of potential sexual transmission receives intense scrutiny, including enquiry into sexual partnership details and travel histories and tests to detect Zika virus in blood, urine, and semen or vaginal secretions. By 9 September 2016, 12 countries had reported cases of non–mosquito-borne Zika virus transmission to the World Health Organization [4], including cases of male-to-female, female-to-male, and male-to-male transmission [5].

Publicly available information about Zika virus infection leaves room for confusion about the relative importance of sex as a route of transmission. The website of the US Centers for Disease Control and Prevention (CDC) lists infected mosquito bites as the primary route of Zika transmission. Transmission through sex is also listed but without any contextual information about the frequency of reported cases, unlike the entries for transmission through blood transfusion or laboratory exposure [6]. The CDC surveillance report shows that only 23 out of 2,382 (1%) reported cases resulted from sexual contact with a traveller to an affected area [7].

Mathematical modelling studies can put the data into context. A Zika-infected person infects on average about two to five additional people when mosquitoes are the vector for transmission [8]. This quantity is the basic reproduction number (*R*_0_), and it can be estimated from the observed incidence of reported cases if we have information on the generation time, which is the average time from infection of one individual to the next individual. In the same setting, estimates of *R*_0_ for Zika are remarkably similar to those for dengue, another mosquito-borne infection, but there is considerable variation among studies done in different countries and populations [9]. The earliest estimates of *R*_0_ for Zika were published before the evidence of sexual transmission started to accumulate. The first modelling study to include this secondary transmission route estimated that sexual transmission contributed about 3.0% to the overall *R*_0_ [10], but there is still considerable uncertainty, with an upper confidence limit of 45.7% in one study [10] and 30% in another [11]. This low point estimate is consistent with the low number of cases in the US that have been attributed to sexual contact with a traveller to an affected area but is still higher than what has been suggested for Ebola [12], for which sexual transmission has been shown to occur only rarely.

Zika is probably not capable of sustained transmission through sexual intercourse in a general population. A prerequisite for a pathogen to spread as a sexually transmitted infection (STI) is that *R*_0_ for sexual transmission, equivalent to the product of the infectious duration and the sexual transmission rate, is greater than one. If the infectious duration of an STI is short (for example, a few months, as is the case with gonorrhoea), transmissibility during a sexual partnership typically needs to be around 50% or more [13]. Of the few cases that have been studied, the longest duration of Zika virus RNA detection in semen is 188 days after the onset of symptoms [14], which is within the range for gonorrhoea. It is unlikely that a transmission probability to sexual partners of around 50% would have gone unnoticed, particularly from individuals with Zika infection who return to countries without endemic circulation.

Investigation with follow up of episodes of sexually transmitted Zika virus infection remains necessary for several reasons. First, Zika could undergo sexual transmission within small clusters involving particular groups with frequent change of sexual partners, such as female sex workers or men who have sex with men. Enhanced surveillance to identify and characterize outbreaks will allow both implementation of control measures and better definition of currently uncertain quantities, including transmission probability and infectious duration. Second, the relative contribution of sexually transmitted cases could increase for populations in which mosquito-borne infections decrease or have not been present at all. Third, viral persistence and sexual transmission could serve as a reservoir for Zika to persist through seasons with low abundance of mosquitoes and could then facilitate regional and international spread. Fourth, larger epidemiological and virological studies are needed to address the many unanswered questions about sexual transmission of Zika virus, such as the prevalence of virus in bodily fluids and the infectivity of persisting virus. A systematic review of studies about sexual transmission of Zika virus would clarify the evidence about all dimensions of the causal relationship, such as temporality, strength of association, and biological plausibility, as has been done for congenital and other neurological complications of Zika [15].

In conclusion, individual case reports show evidence that Zika virus has been transmitted from person to person through sexual intercourse—so it is sexually transmissible. But sexual transmission is not the primary route of transmission. For a disease to be designated a sexually transmitted infection, sexual intercourse should account for a substantial proportion of infections. Information and advice for the public should give clear messages about the relative contributions of mosquito-borne, vertical, sexual, and bloodborne transmission so that people can make informed choices about the preventive measures that they should take.
